# Optimal Design and Analysis of Wide-Band Near-Infrared Hybrid Dielectric Gratings with High Transmission Efficiency

**DOI:** 10.3390/mi15111290

**Published:** 2024-10-23

**Authors:** Ye Wang, Yongyi Chen, Li Qin, Lijun Wang

**Affiliations:** 1 Key Laboratory of Luminescence Science and Technology, Chinese Academy of Sciences, Changchun 130033, China; wyoptics@163.com (Y.W.); qinl@ciomp.ac.cn (L.Q.); 2 State Key Laboratory of Luminescence Science and Applications, Changchun Institute of Optics, Fine Mechanics and Physics, Chinese Academy of Sciences, Changchun 130033, China; 3 Peng Cheng Laboratory, No. 2, Xingke 1st Street, Nanshan, Shenzhen 518000, China; 4 Jlight Semiconductor Technology Co., Ltd., No.1588, Changde Road, Economic Development Zone, Changchun 130102, China; 5 Academician Team Innovation Center of Hainan Province, Key Laboratory of Laser Technology and Optoelectronic Functional Materials of Hainan Province, School of Physics and Electronic Engineering, Hainan Normal University, Haikou 570206, China

**Keywords:** near-infrared, transmission grating, multilayer dielectric, fabrication tolerance

## Abstract

Since surface relief transmission gratings have very strict requirements on operators and use environment, according to the semiconductor laser external cavity spectral beam combining system, this paper proposes a design scheme for a semiconductor laser array spectral beam combining system based on the grating-external cavity. The finite element approach was used to create a wideband, high-efficiency fill-in multilayer dielectric transmission grating structure for a high-power spectrum beam combining system. The incidence angle, ridge height, duty cycle, and sidewall inclination angle of the transmission grating were tuned and evaluated, and a link between the transmission grating’s diffraction efficiency and grating characteristics was discovered. The calculated design of the high-power fused silica transmission grating has a negative first-order peak diffraction efficiency of 99.5% in the 800 nm range. In the spectral region of 765–872 nm, the transmission grating’s diffraction effectiveness exceeds 92%. The filled ultra-high diffraction efficiency multilayer dielectric transmission grating design addresses the issue of resistance to high-power lasers under complicated operating settings. It is intended to maintain a high diffraction efficiency even after several cleaning cycles, and it is an ideal component for high-power spectrum beam combining systems.

## 1. Introduction

Near-infrared transmission gratings at 800 nm have garnered significant attention due to their unique application potential. Systems for laser spectrum synthesis, wavelength modulation, and spectral detection must integrate with high-power near-infrared (NIR) laser systems [1]. Each system’s performance improves, as does the performance of multilayer dielectric gratings, allowing for the development of a variety of laser systems and designs. Diffraction gratings are considered the weakest fundamental components within high-power laser systems and are crucial for achieving high-power spectral beam combinations. The diffraction efficiency of these gratings directly influences the output energy and beam quality of the high-power laser system, thereby ensuring the system’s ability to maintain high performance over an extended service life [2,3].

Multilayer dielectric film gratings are frequently utilized because dielectric film absorption is extremely low and may be ignored, and the anti-laser damage threshold is many orders of magnitude larger than that of metal gratings [4]. The laser output is diffracted four times in the spectral beam combining system, and the comprehensive diffraction efficiency is η^4^ (η is the diffraction efficiency of the grating). As a result, increasing the grating’s DE from 94.5% [5] for ordinary metal grating to 99.5% will ultimately increase the comprehensive diffraction efficiency by 18.27%. Moreover, the transmission grating is better than the reflective grating because the optical elements are easier to adjust. The research presented in this study focuses on filled hybrid dielectric transmission gratings with excellent diffraction efficiency and a high damage resistance threshold.

Using only dielectric materials, an optimized grating structure was reported with 40 nm bandwidth around 800 nm wavelength, meaning that the DE spectrum showed higher than 97% efficiency between 780 and 820 nm [6]. In this article, a double-polished fused silica substrate is utilized to model a rectangular, filled transmission grating appropriate for an SBC system in the near-infrared band using the finite element analysis method. The diffraction level of the grating is adjusted by gradient simulation and optimization of the grating structure, as well as technical parameters such as period, groove depth, and duty cycle, so that the grating’s diffraction energy is concentrated in the first diffraction stage and the grating’s diffraction efficiency is more than 92% in the working spectral range of nearly 100 nm. This work not only provides a novel approach for developing transmission gratings with high diffraction efficiency and a large operating spectral range, but it also gives a new method for guiding the grating production process. The newly filled multilayer dielectric transmission grating is suitable for semiconductor laser array external cavity feedback spectrum beam combining to obtain high power, high power density, and high beam quality laser output.

## 2. Optimal Multilayer Dielectric Design in Grating Grooves

**Figure 1:**
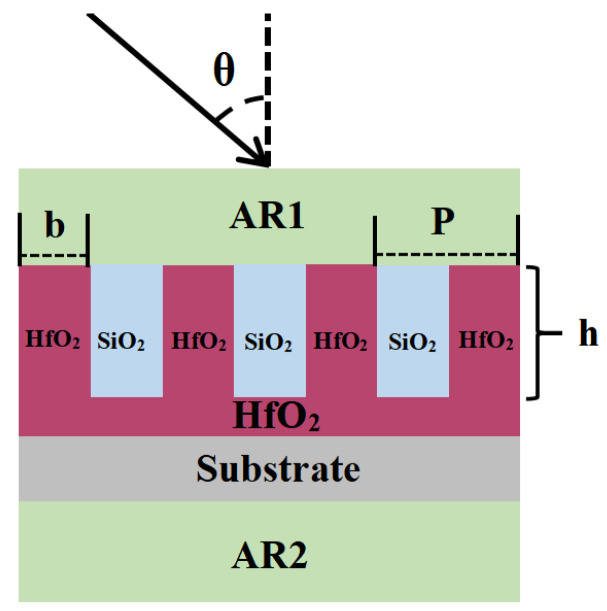
Schematic diagram of 800 nm filled multilayer dielectric transmission grating structure.

Figure 1 illustrates the structural diagram of an 800 nm filled-in multilayer transmission grating. The grating layer and the anti-reflection coating are designed independently. The primary objective of the grating layer design is to achieve a broad and high diffraction efficiency. This is accomplished by optimizing the refractive index difference between the interfaces, such as those between the grating layer and air, between the quartz substrate and the underlying HfO_2_ layer, and between the quartz substrate and air. As shown in Figure 1, the beam is lighted by a Littrow angle beam from air with a refractive index of 1.0. From the top layer to the bottom layer, the multilayer dielectric transmission grating comprises an anti-reflective coating AR1, a grating layer, an etching barrier layer, a fused silica substrate, and an anti-reflective coating AR2, where the depth of the filling material layer is h, and the filling material layer can protect the grating surface from high laser damage and pollution; The width of the ridge is b, and the period is P. The grating duty cycle ff is defined as the ratio of grating ridge width to grating period, i.e., ff = b/p. The diffracted beam is transmitted from the grating’s bottom via a sequence of light matter diffraction processes. This study focuses on the fundamental diffraction order.

According to the finite element theory, the diffraction efficiency of the grating depends on the geometry parameters of the grating under the conditions of a specific wavelength, polarization state, and angle of incidence, and with proper geometric parameter tuning, the NIR transmission grating’s near-diffraction limit can approach 100%. The basic grating specifications are as follows: the center wavelength is 800 nm, the line density is 1800 line/mm, accordingly the incidence angle is 46.6°. The goal of grating design is to produce the maximum feasible diffraction efficiency over a wide spectral range. DE is greater than 92 percent exceed the width of 0.1 μm. The shape of the grating is set to be a rectangular groove. These parameters are listed in Table 1 below.

### 2.1. Transmission Grating Based on Pure Fused Silica and Si_3_N_4_ Grating

**Figure 2:**
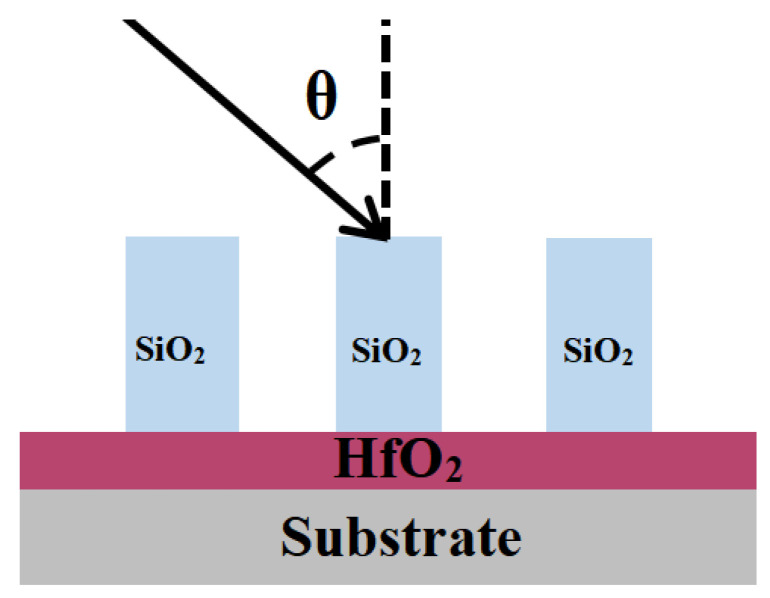
Schematic diagram of fused silica grating or Si_3_N_4_ grating structure of air groove.

**Figure 3:**
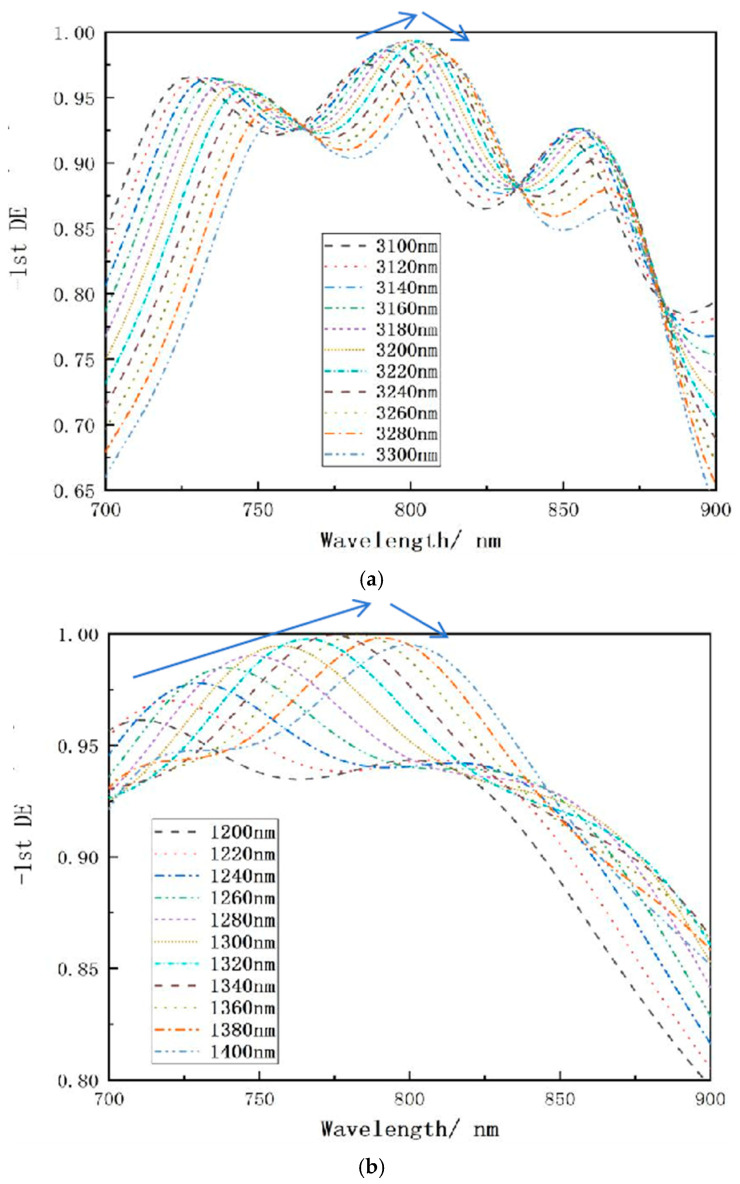
Comparison of diffraction efficiency between fused silica grating and Si_3_N_4_ grating. (**a**) Relationship between wavelength and diffraction effectiveness at various groove depths of air groove’s fused silica grating. (**b**) The relationship between wavelength and diffraction efficiency at varying groove depths of the Si_3_N_4_ grating.

The air groove’s fused silica and Si_3_N_4_ gratings were simulated, and Figure 2 depicts the schematic construction. The period and duty cycle of the grating are consistent with those listed in Table 1 of this document. The air groove’s fused silica grating requires a larger grating depth (~3.2 μm) to attain equivalent diffraction efficiency as the Si_3_N_4_ grating (~1.36 μm); however, etching 3.2 μm fused silica needs an etching technique with a higher aspect ratio than 1:11, compared to Si_3_N_4_, the encoder only requires an aspect ratio of around 1:5, therefore, Si_3_N_4_ gratings are easier to fabricate, and it should be noted that the diffraction efficiency of Si_3_N_4_ gratings at the working wavelength is around 92%, as seen in Figure 3a,b.

### 2.2. Using HfO_2_ Filled Instead of Pure SiO_2_ for the Grating

**Figure 4:**
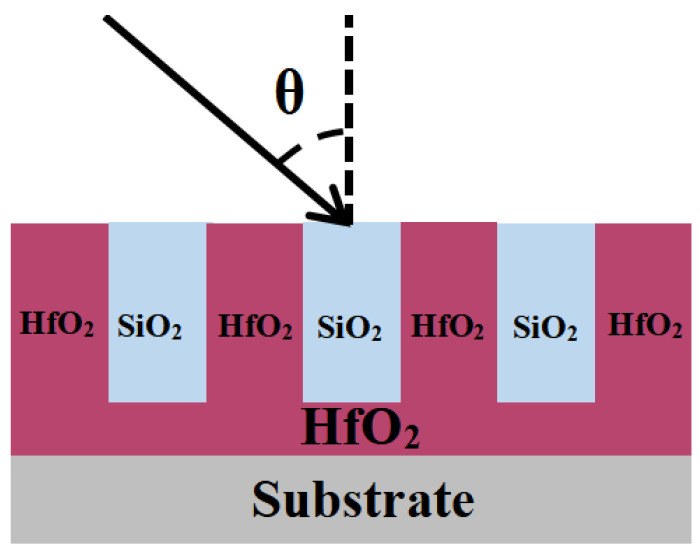
Hybrid grating buried in SiO_2_ material.

As analyzed in Section 2.1, the theoretical design of a transmission grating with a pure quartz structure predicts a maximum diffraction efficiency (DE) of around 92%, which is not high enough for the high-power spectral beam combination system. Meanwhile, this structure is difficult to manufacture because of its high etching depth of over 3 μm. Figure 4 shows how the whole grating layer is filled with HfO_2_ material to maximize the maximum DE values and accessible spectral range.

Table 1 expresses the background refractive index as the air refractive index, and the refractive index difference is derived based on the substrate material. The substrate was fused silica with a refractive index of 1.58; hence, the refractive index difference was 0.58. To make the simulation results more realistic, a 0.5 mm size with the same thickness as the actual quartz sheet was used for simulation. The simulation procedure focuses on the impact of the grating’s structural characteristics (incident angle, duty cycle, groove depth, and sidewall inclination angle) on diffraction efficiency, with the control variable technique employed for optimization analysis.

#### 2.2.1. The Effect of Groove Depth

The major goal of the fill-in multilayer dielectric transmission grating is to provide an appropriate grating groove depth, given that, as the grating etching depth grows, the etching process complexity increases exponentially. Furthermore, as the etching depth grows, so does the inclination angle of the grating sidewall, and the etching pattern eventually takes on a trapezoidal form, which has a significant impact on the grating’s diffraction efficiency. As a result, the shorter the simulation range for groove depth while preserving grating diffraction effectiveness, the more practical it is.

**Figure 5:**
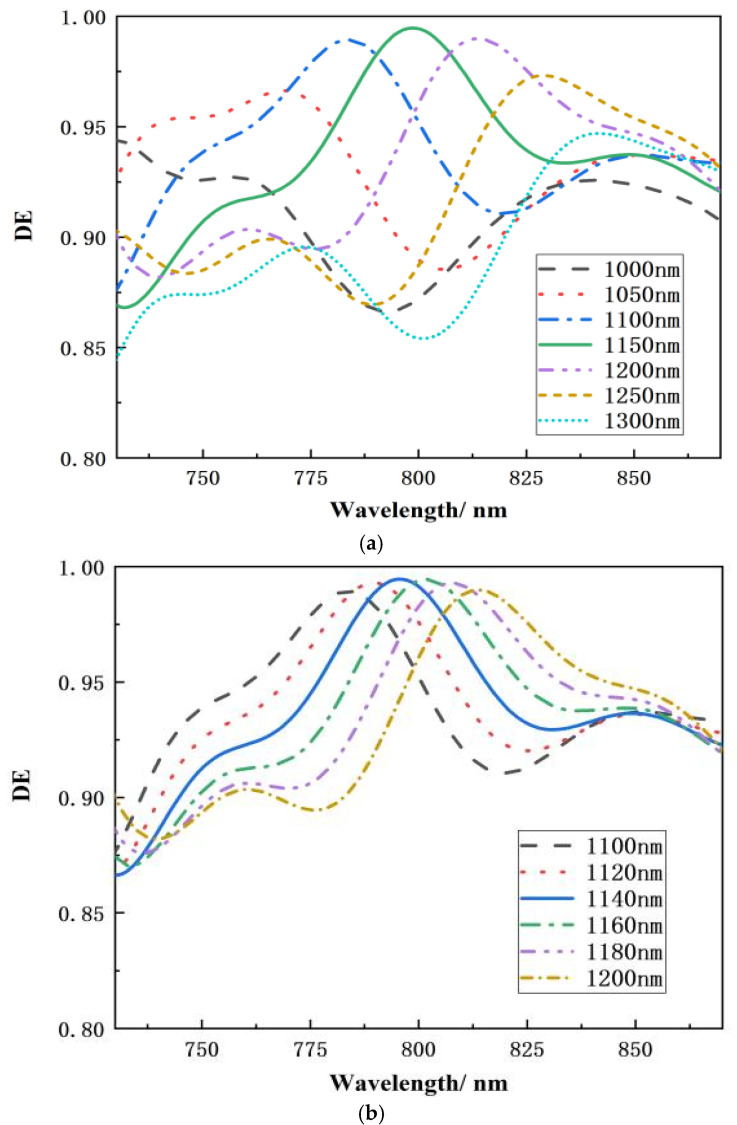
Relationship between wavelength and diffraction efficiency at various grating groove depths. (**a**) Depth of 1000–1300 nm with 50 nm spacing. (**b**) Depth of 1100–1200 nm with 20 nm intervals.

According to the simulation analysis of different grating groove depths in Figure 5a, it can be seen that the diffraction efficiency of the grating is higher when the grating groove depth is in the range of 1.1–1.2 μm. The challenge in this study is to employ HfO_2_ to fill the SiO_2_ grating groove, which necessitates precise measurement of the grating groove depth and setting the step interval to 20 nm, as illustrated in Figure 5b, during all simulations, the direction of polarization is perpendicular to the paper. When the depth of the grating groove is reduced, the overall spectral curve gets bluer and the peak diffraction efficiency reduces significantly. As the grating groove depth increases, the overall spectral curve redshifts, and peak diffraction efficiency diminishes when the incidence angle is constant and the groove depth is 1160 nm, the transmission grating’s diffraction efficiency at the operating wavelength exceeds 99 percent.

**Figure 6:**
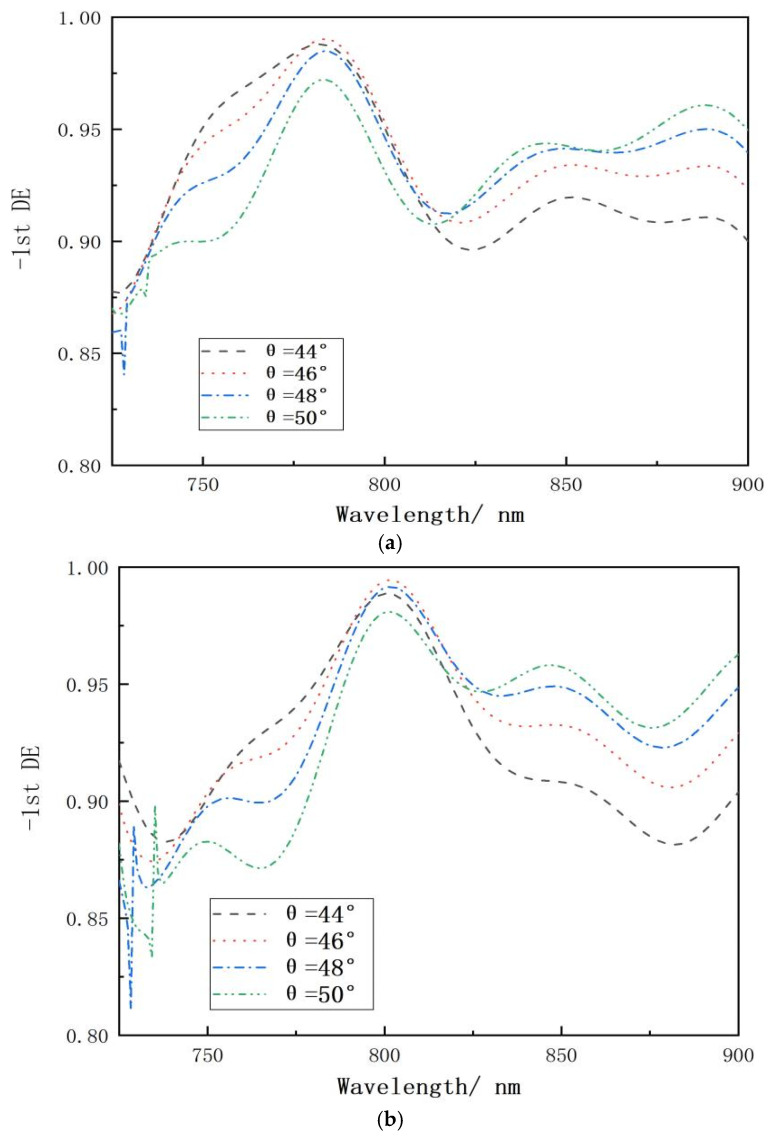

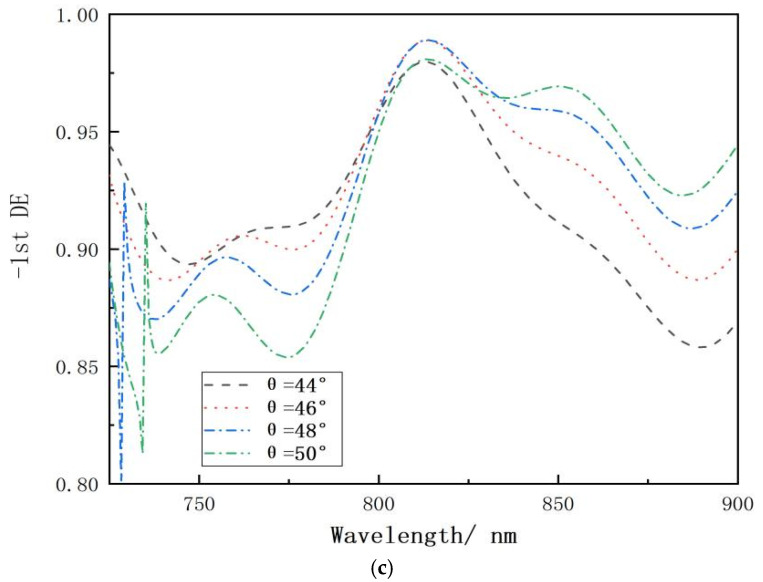
Relationship between wavelength and diffraction effectiveness at various groove depths and angles of incident. (**a**) When the grating groove depth is 1100 nm. (**b**) When the grating groove depth is 1160 nm. (**c**) When the grating groove depth is 1200 nm.

When the grating groove depth is 1100 nm, the overall spectral curve shifts blue at various incidence angles, as seen in Figure 6a. When the grating groove depth is 1160 nm and the incidence angle is 46°, the peak diffraction efficiency at the center wavelength of 800 nm is 99.5%, while the diffraction efficiency throughout the 100 nm bandwidth region is more than 93%, as shown in Figure 6b. When the grating groove depth is 1200 nm, the entire spectral curve is redshifted at all angles of incidence, as seen in Figure 6c.

**Figure 7:**
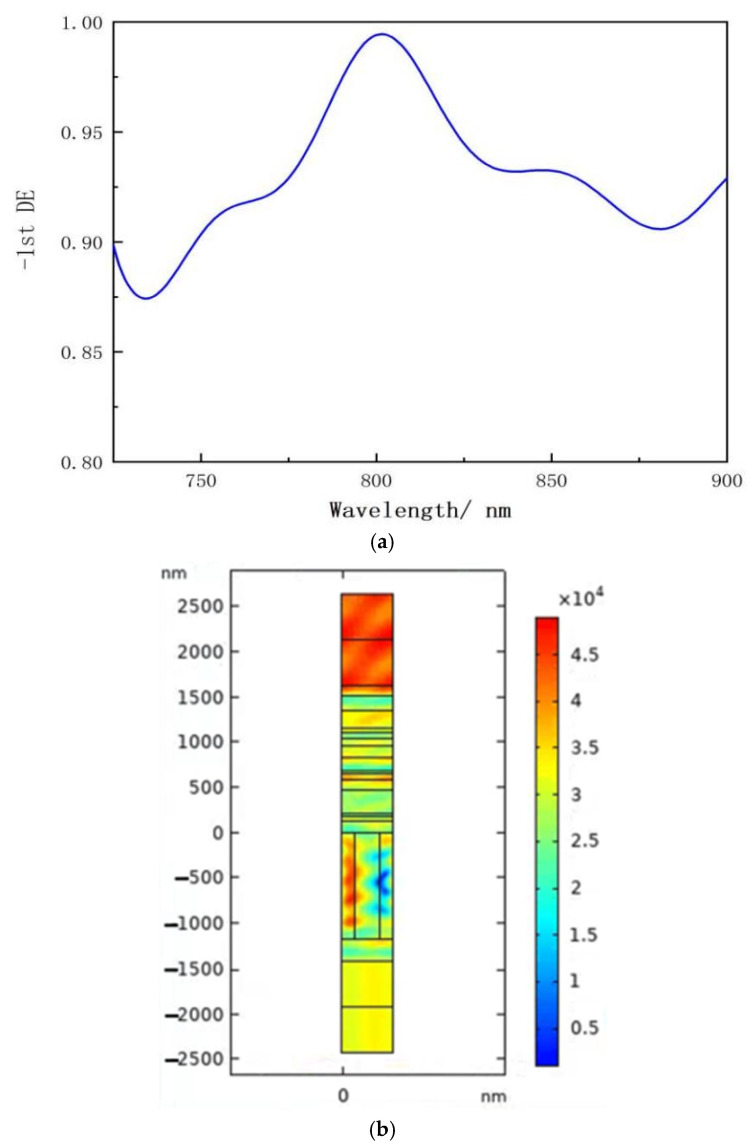
(**a**) MDTG DE with a grating groove depth of 1160 nm and an incidence angle of 46°; (**b**) MDTG electric field intensity distribution with an incidence angle of 46° and grating groove depth of 1160 nm.

The high-power SBC system has an operational spectral range of roughly 100 nm. When the angle of incidence is 46° and the depth of the grating groove is 1160 nm, as illustrated in Figure 7a, the multilayer dielectric transmission grating has a higher diffraction efficiency than the metal grating. The laser-induced damage threshold for high-power diode lasers is higher because the effects of intrinsic absorption of metallic materials are eliminated, and the simulated electric field distribution is shown in Figure 7b.

#### 2.2.2. The Effect of Incidence Angle

The angle scan demonstrates that by fine-tuning the angle under the given period, grating groove depth, and duty cycle, the diffraction efficiency can be close to 100%, increasing the process tolerance of the preparation grating and making it easier to adjust the position of the diffraction grating and achieve high-power laser output in actual spectral beam combination operation.

**Figure 8:**
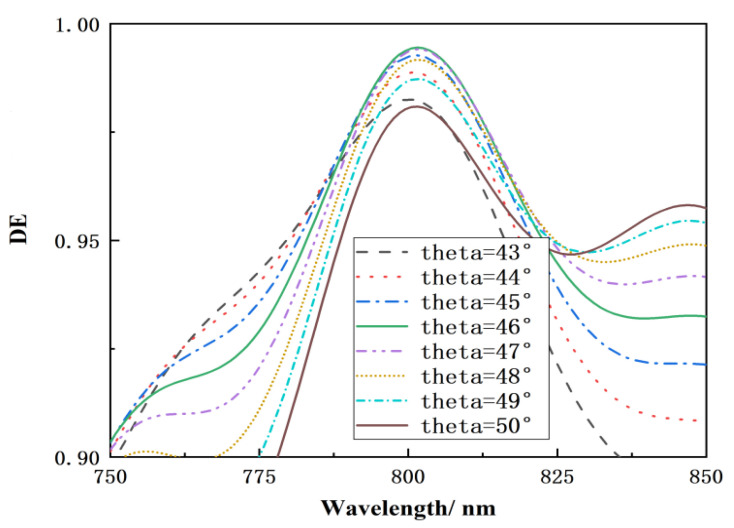
The relationship between wavelength and diffraction efficiency at various angles of incidence.

Figure 8 shows that when the incidence angle is 43°–50°, the fill-flat multilayer dielectric film transmission grating has a diffraction efficiency of more than 98% at the working wavelength of 800 nm. When the incidence angle is less than 46°, the peak diffraction efficiency declines, as does the diffraction efficiency in the region above 800 nm. When the incidence angle exceeds 46°, the band less than 800 nm diminishes significantly, while the diffraction effectiveness of the band bigger than 800 nm rises. The design accomplishes the intended function by focusing energy on the first transmission stage, with no energy in the 0th diffraction stage at 800 nm. The near-infrared multilayer dielectric transmission grating has a high diffraction efficiency within a certain range of incoming angles, giving it some flexibility in the design of a near-infrared high-power laser SBC system.

#### 2.2.3. The Effect of Duty Cycle

**Figure 9:**
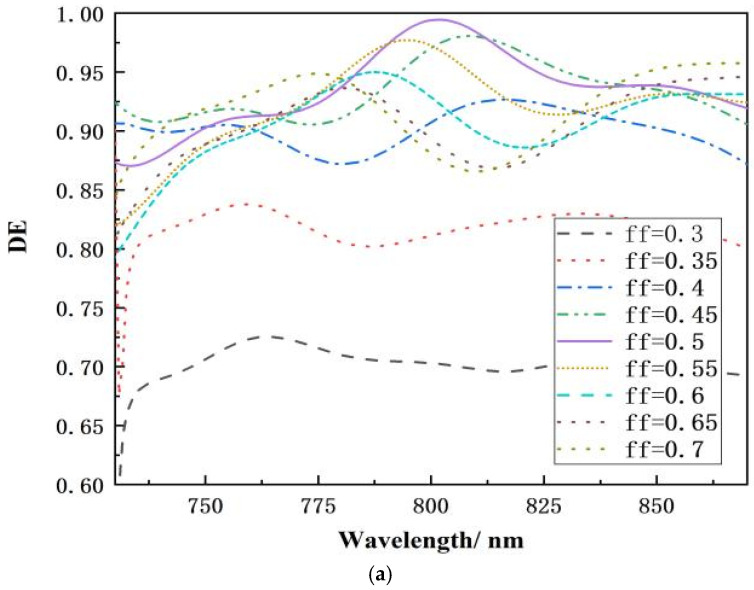

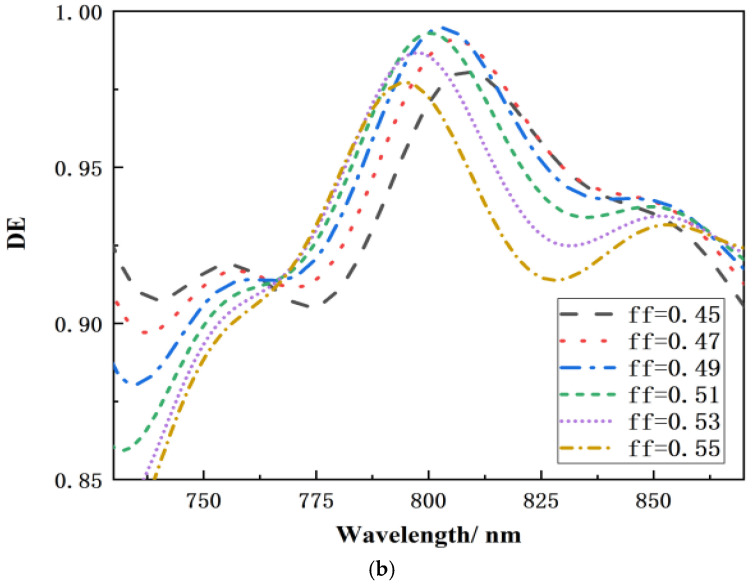
Relationship between wavelength and diffraction efficiency at various duty cycles: (**a**) 0.3–0.7 with intervals of 0.05; (**b**) 0.45–0.55 with intervals of 0.05.

The grating’s duty cycle has a significant impact on its diffraction efficiency, and the diffraction efficiency of different duty cycles varies greatly for transmission gratings. Figure 9a shows a simulation of a filling factor (ff) ranging from 0.3 to 0.7, with a step interval of 0.05. To produce an accurate duty cycle, the duty cycle interval was adjusted at 0.45–0.55, with a step interval of 0.02 for gradient simulation. When the grating duty cycle is between 0.48 and 0.52, the peak diffraction efficiency at the 800 nm band is around 99%, which serves as a valuable reference for grating manufacture with substantial process tolerances, as seen in Figure 9b.

**Figure 10:**
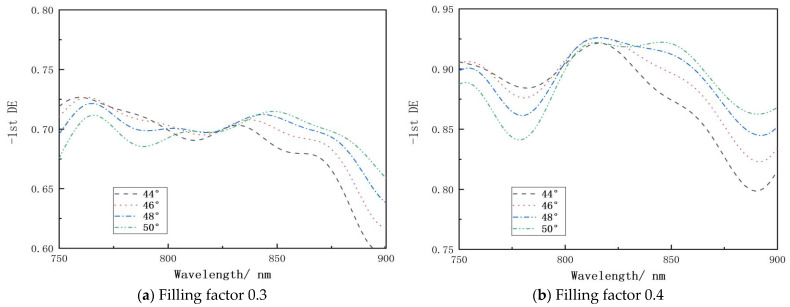

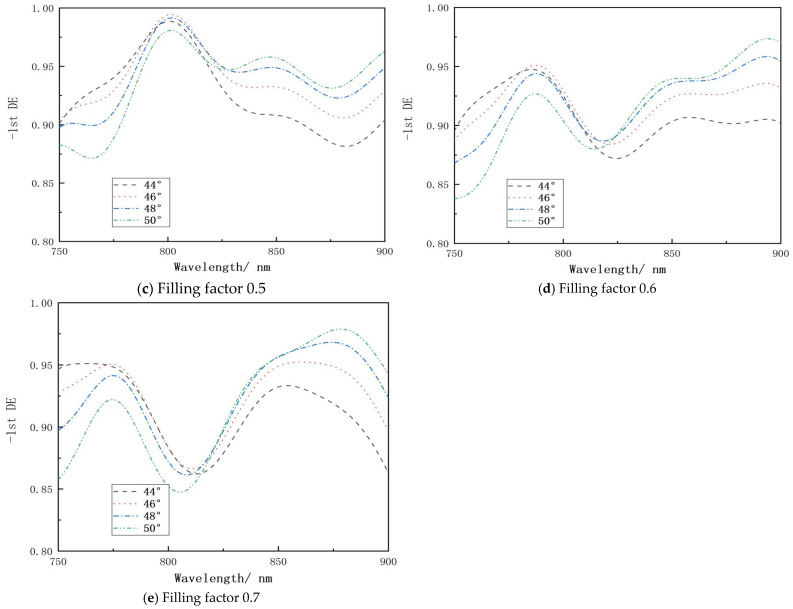
The relationship between wavelength and diffraction efficiency at various duty cycles and angles of incidence: (**a**) filling factor 0.3; (**b**) filling factor 0.4; (**c**) filling factor 0.5; (**d**) filling factor 0.6; (**e**) filling factor 0.7.

The incident light has a wavelength of 800 nm and an angle of incidence of 46.6°. In the actual production process, there are inherent process defects in the width of the groove, which primarily affect the duty cycle and then affect the diffraction efficiency; the question is how significant the effect is and whether it is acceptable. The grating groove depth was set to 1160 nm, and the effects of different angles of incidence on grating performance under various duty cycles were simulated, as shown in Figure 10. The diffraction efficiency of a grating at the central wavelength is significantly influenced by its duty cycle. Typically, when the duty cycle is less than 0.5, the efficiency is less than 90%. Optimally, at a duty cycle of 0.5, the peak diffraction efficiency can approach 100% at a specific incidence angle. However, a duty cycle exceeding 0.5 also tends to result in reduced efficiency. This analysis of the relationship between duty cycles and incidence angles is crucial for guiding the design of gratings in future experiments, aiming to achieve high diffraction efficiency.

Compared to the air groove’s pure quartz transmission grating, the filled multilayer dielectric transmission grating has a maximum diffraction efficiency of 99.5%, and the grating trough is very simple to build.

#### 2.2.4. The Effect of Sidewall Inclination

**Figure 11:**
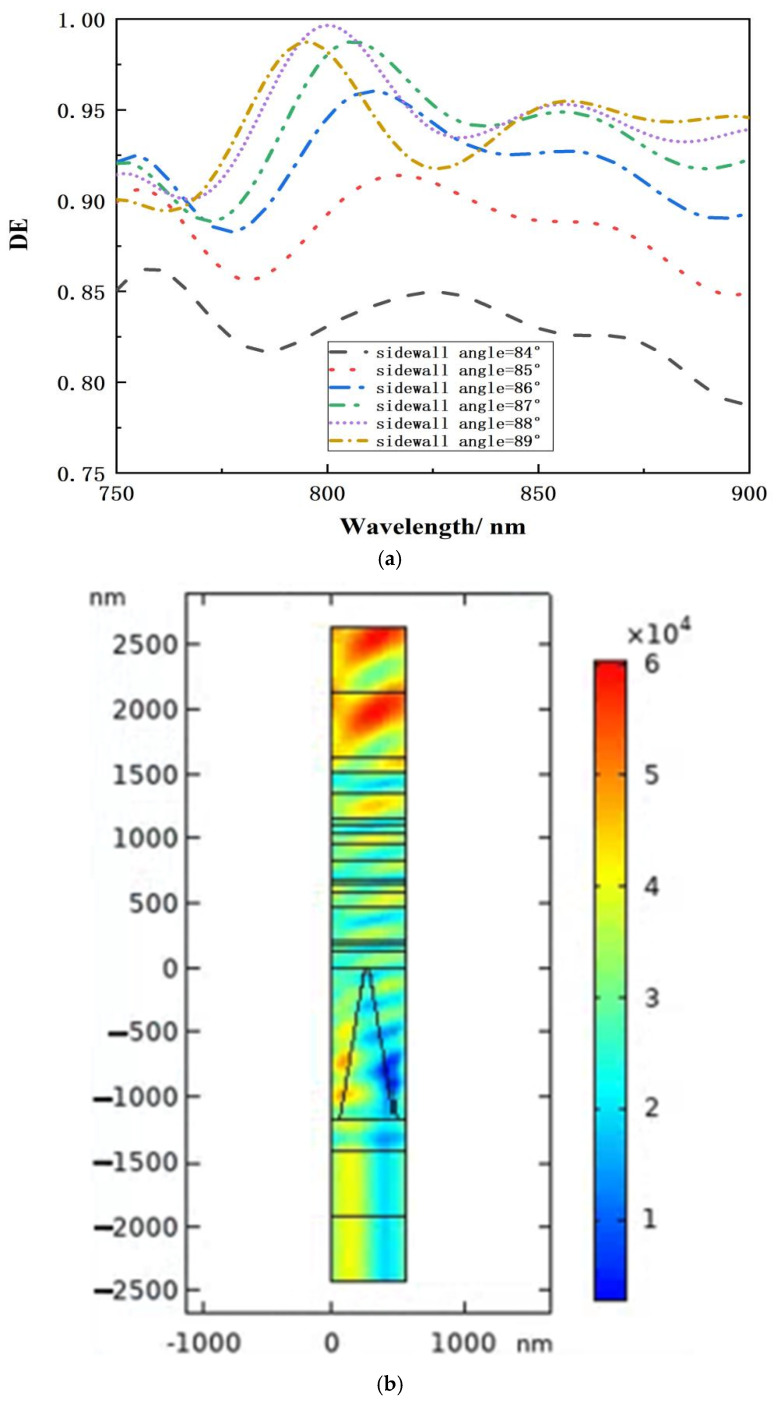

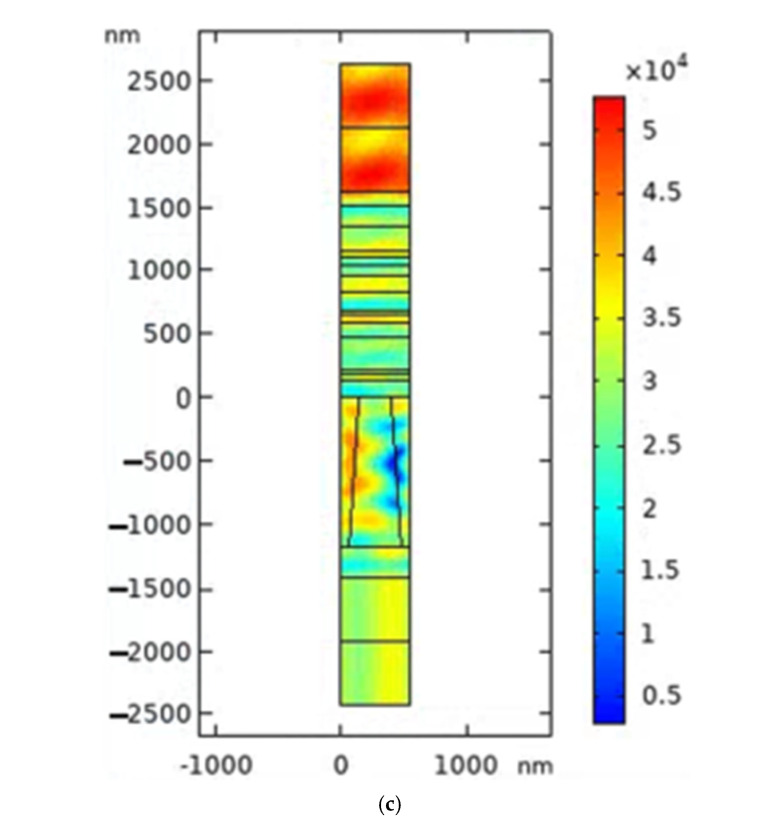
Diffraction efficiency and EFI enhancement effects of MDTG at various sidewall inclination degrees. (**a**) Relationship between wavelength and diffraction effectiveness at various sidewall inclination degrees. (**b**) Field strength distribution at sidewall inclination angle of 84°. (**c**) Field strength distribution at sidewall inclination angle of 89.5°.

In the typical production process, the shape of the fill-flat multilayer dielectric film transmission grating structure degenerates into a trapezoidal topography, affecting MDTG’s diffraction efficiency and near-field EFI enhancement. The sidewall angle has been described as the angular divergence between the grating sidewall and the coated surface ranging from 90° to 70° [7]. Figure 11 depicts the diffraction efficiency and EFI enhancement effects of MDTG at various sidewall inclination degrees. The electric field intensity distribution diagram shows that the maximum EFI in the ridge of the 84° trapezoidal grating is similar to that of the 90° rectangular grating structure, implying that the structure’s filled transmission grating is not significantly different in terms of laser damage resistance. As demonstrated in Figure 11a, the diffraction efficiency of MDTG at the operating wavelength is best when the angle is near 90 degrees. When the side wall of the grating is inclined at 84°, the grating’s overall diffraction performance deteriorates. As a result, controlling the MDTG as a rectangular profile is worth paying a higher price.

### 2.3. Anti-Reflective Coating Design

AR coating technology may be used on the surface of the line grating to raise the anti-laser damage threshold of MDTG while also reducing reflection [8]. To accomplish destructive interference of reflected light, the anti-reflective coating’s effective refractive index should be equal to that of the quartz, or a phase-matched layer should be put between the fused silica and the anti-reflective coating. This allows for a phase difference of π between two neighboring reflected light beams. With this design, reflected light may be effectively minimized. This AR coating procedure is particularly appropriate for transmission gratings.

The substrate material of 800 nm MDTG is fused silica, and the dielectric film materials are HfO_2_, Ta_2_O_5_, and SiO_2_. HfO_2_ and Ta_2_O_5_ are high-refractive-index materials that withstand laser damage. SiO_2_, a material with a low refractive index, is very resistant to laser damage due to its material features [9]. When calculating optical performance in this operational spectral range, the coating’s absorption and refractive index dispersion with wavelength are neglected. The anti-reflective coating was created with TFC, a specialist thin film design program, and the diffraction efficiency of TE and TM was kept as near to one as feasible in the operational wavelength range using the Littrow configuration. The original film environment settings were set to 800 nm @ 800 nm and 1800 lines/mm of kern density. As a consequence, with an angle of incidence or Littrow angle of 46.6° and an incident medium of air, AR coatings with an uneven film structure will provide better manufacturing flexibility.

By employing the transmission matrix theory to optimize the thickness and refractive index of each stratum within the antireflection coating, it is possible to achieve superior transmittance across the operational bandwidth. The matrix expression for a single layer is

(1) $$\left(\begin{array}{c} B\\ C \end{array}\right)=\left(\begin{array}{cc} cos\delta_{1} & \frac{i}{\eta_{1}}sin\delta_{1}\\ i\eta_{1}sin\delta_{1} & cos\delta_{1} \end{array}\right)\left(\begin{array}{c} 1\\ \eta_{2} \end{array}\right)$$

(2) $$\text{in}\;\;\;\;\;\left(\begin{array}{cc} cos\delta_{1} & \frac{i}{\eta_{1}}sin\delta_{1}\\ i\eta_{1}sin\delta_{1} & cos\delta_{1} \end{array}\right)$$

The film’s useful parameters are all contained in what is known as the characteristic matrix of the film. From the substrate and film’s characteristic matrix, Y = C/B, one may derive the combined admittance.

Similar results can be derived for the multilayer film in terms of the characteristic matrix of the film system:

(3) $$\left(\begin{array}{c} B\\ C \end{array}\right)=\prod_{j=1}^{k}\left(\begin{array}{cc} cos\delta_{1} & \frac{i}{\eta_{1}}sin\delta_{1}\\ i\eta_{1}sin\delta_{1} & cos\delta_{1} \end{array}\right)\left(\begin{array}{c} 1\\ \eta_{k+1} \end{array}\right)$$

(4) $$\delta_{j}=\frac{2\pi }{\lambda }N_{j}d_{j}cos\theta_{j}$$

$$\left(\begin{array}{cc} cos\delta_{1} & \frac{i}{\eta_{1}}sin\delta_{1}\\ i\eta_{1}sin\delta_{1} & cos\delta_{1} \end{array}\right)$$

is the feature matrix in the jth layer. The transmittance and absorption of the film system can be calculated by Equation (3) [10]. Table 2 lists the film thickness of AR1 and AR2.

**Figure 12:**
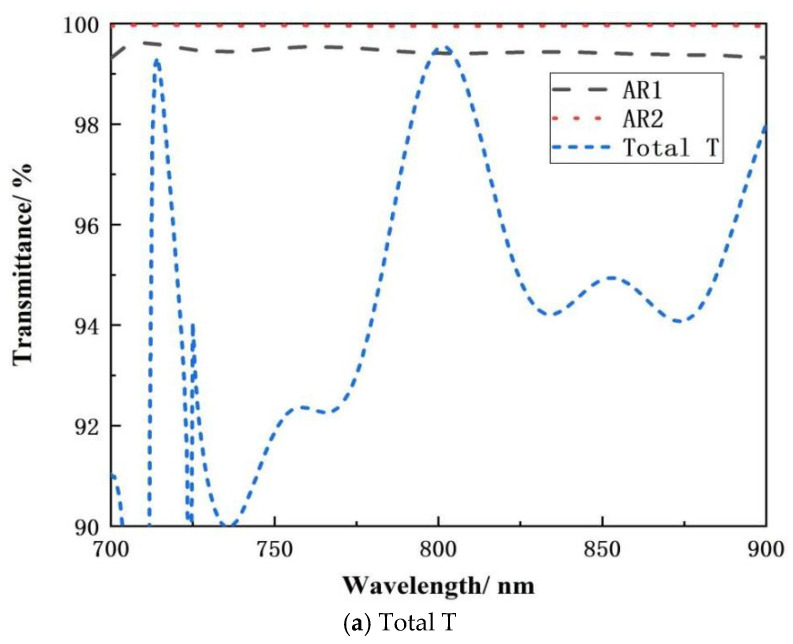

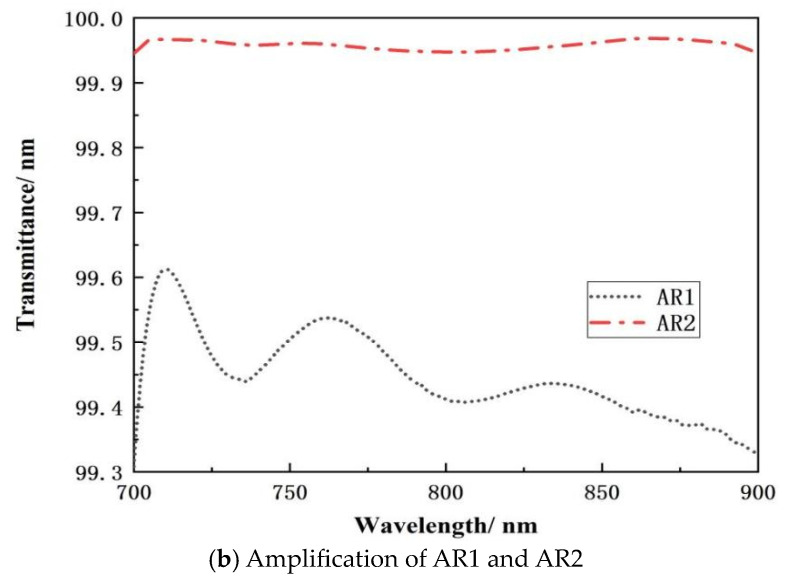
Spectral transmittance in the near-infrared band, (**a**) total T; (**b**) amplification of AR1 and AR2.

**Figure 13:**
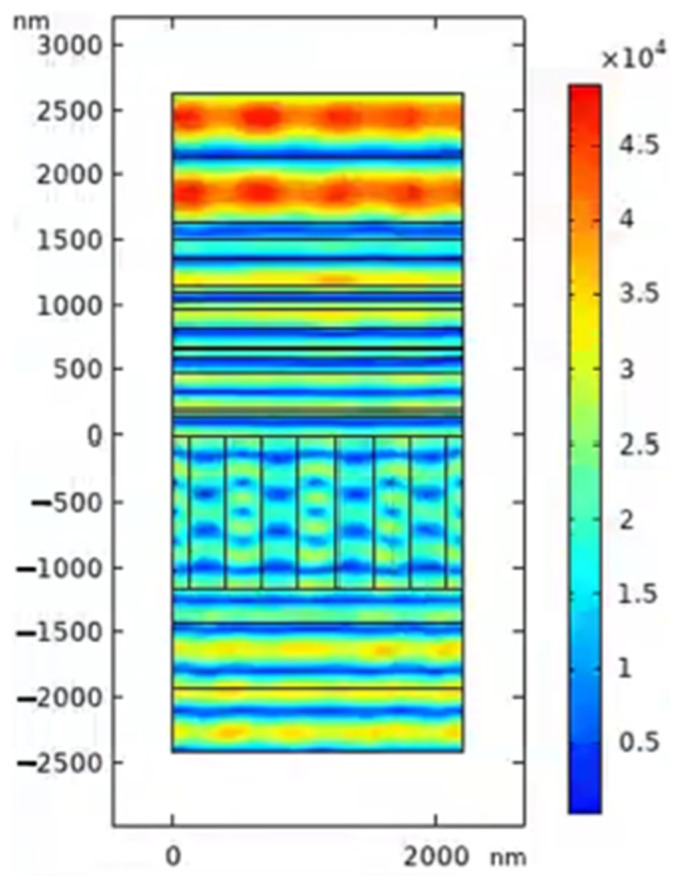
Total electric field intensity distribution of near-infrared MDTG.

Figure 12 displays the theoretical design spectral curves of the anti-reflective coatings AR1 and AR2 for the structure illustrated in Figure 1. Figure 12 shows that the AR1 thin film structure has an average transmittance of 99.5% between 700 and 900 nm. The AR2 thin film structure has an average transmittance of 99.95% in the 700–900 nm spectral region. At 100 nm bandwidth, the average spectral transmittance exceeds 99%, meeting the design and application criteria for near-infrared transmission gratings. To optimize the study, the developed anti-reflective coating AR1 and AR2 film structures were included in the Comsol Multiphysics simulation tool. Figure 12 depicts the transmission spectrum of the total grating structure, with the grating layer structure and anti-reflective coating properly matched. Figure 13 shows the total field strength distribution of the 800 nm filled multilayer dielectric transmission grating.

## 3. Conclusions

Metal gratings’ intrinsic absorption results in low diffraction efficiency and anti-laser damage thresholds. This study proposes a fill-in multi-layer dielectric film transmission grating with four areas: a substrate, a fill-in layer, an anti-reflective coating, and an etching barrier layer. According to the optimization findings, the maximum diffraction efficiency is more than 99.5% at 800 nm, the working spectral range is more than 100 nm, and the lowest diffraction efficiency is more than 92% in the 765–872 nm band (grating period 556 nm, incidence angle 46.6°, duty cycle 0.5). The optimized design of the new fill-in multilayer dielectric transmission grating can increase the output power of the laser SBC system, provide a practical technology for improving the power and brightness of the SBC, and maintain a high diffraction efficiency even after multiple cleaning cycles; it ensures the efficient application of the ultra-high diffraction efficiency grating. Future research will concentrate on large-area near-infrared multilayer transmission gratings, including manufacturing technology, testing methods, and application verification in high-power SBC systems.

## Figures and Tables

**Table 1:** Transmission grating parameters.

Spectral Range	750–850 nm
Incidence angle	46.6°
Substrate	Fused Silica
Period	566 nm
Filling Factor	0.5
Background refractive index	1

**Table 2:** Design results of the optimized AR coating system.

**(a)** **AR1 Coating System.**					
**AR1, Layer NO.**	**Layer Material**	**Layer Thickness/nm**	**AR1, Layer NO.**	**Layer Material**	**Layer Thickness/nm**
Incidence angle	46°		7	SiO_2_	148
Incident Medium	Air		8	Ta_2_O_5_	138
1	SiO_2_	38	9	SiO_2_	71
2	Ta_2_O_5_	38	10	Ta_2_O_5_	64
3	SiO_2_	261	11	SiO_2_	50
4	Ta_2_O_5_	118	12	Ta_2_O_5_	208
5	SiO_2_	62	13	SiO_2_	149
6	Ta_2_O_5_	27	Exit Medium	HfO_2_	
**(b)** **AR2 Coating System.**					
**AR1, Layer NO.**	**Layer Material**	**Layer Thickness/nm**	**AR1, Layer NO.**	**Layer Material**	**Layer Thickness/nm**
Incidence angle	29°		14	SiO_2_	143.6
Incident Medium	Air		15	Ta_2_O_5_	68.91
1	Ta_2_O_5_	23.1	16	SiO_2_	75.91
2	SiO_2_	99.51	17	Ta_2_O_5_	247.29
3	Ta_2_O_5_	62.37	18	SiO_2_	382.4
4	SiO_2_	96.41	19	Ta_2_O_5_	214.99
5	Ta_2_O_5_	59.17	20	SiO_2_	115.57
6	SiO_2_	108.38	21	Ta_2_O_5_	46.53
7	Ta_2_O_5_	74.62	22	SiO_2_	41.72
8	SiO_2_	104.62	23	Ta_2_O_5_	65.42
9	Ta_2_O_5_	41.63	24	SiO_2_	270.9
10	SiO_2_	115.69	25	Ta_2_O_5_	106.53
11	Ta_2_O_5_	187.34	26	SiO_2_	137.02
12	SiO_2_	96.08	27		
13	Ta_2_O_5_	21.07	Exit Medium	Fused Silica	

## Data Availability

The original contributions presented in the study are included in the article, further inquiries can be directed to the corresponding authors.
